# Hepatitis A virus knowledge and immunization attitudes and practices in the United Arab Emirates community

**DOI:** 10.1038/s41598-020-80089-4

**Published:** 2021-01-29

**Authors:** Kamel A. Samara, Hiba J. Barqawi, Basant H. Aboelsoud, Moza A. AlZaabi, Fay T. Alraddawi, Ayten A. Mannaa

**Affiliations:** 1 College of Medicine, University of Sharjah, Sharjah, United Arab Emirates; 2 Department of Clinical Sciences, College of Medicine, University of Sharjah, Sharjah, United Arab Emirates

**Keywords:** Infectious diseases, Viral hepatitis, Epidemiology, Preventive medicine, Health policy, Public health

## Abstract

Annually, 1.5 million cases of hepatitis A infection occur worldwide. The United Arab Emirates (U.A.E.) has seen a decrease in infection rates and seroprevalence coupled with an increase in the average age of infection. This study aimed to assess the U.A.E. society’s hepatitis A knowledge, and attitudes and vaccination practices, with the applicability of its introduction into the local immunization schedule. A self-administered, 50-item questionnaire was used to collect data from the four most populous cities in the U.A.E., between January and March 2020. A total of 458 responses were collected and analysed using IBM-SPSS-26, R-4.0.0 and Matplotlib-v3.2.1. Females had better attitudes (P = 0.036), practices (P < 0.0005), immunization schedule knowledge (AOR = 3.019; CI 1.482–6.678), and appreciation of the immunization schedule (AOR = 2.141; CI 1.310–3.499). A higher level of perceived knowledge was associated with an actual better knowledge (P < 0.0005), better practices (P = 0.011), and increased willingness to get vaccinated (AOR = 1.988; CI 1.032–3.828). Respondents were more likely to vaccinate their children against HAV if the vaccine were introduced into the National Immunization Program (P < 0.0005). Overall, disease knowledge was lacking but with positive attitudes and poor practices. There is high trust in the National Immunization Program and a potential for improving poor practices through local awareness campaigns.

## Introduction

Hepatitis A Virus (HAV) is a small, quasi-enveloped, single-stranded, positive RNA virus belonging to Picornaviridae and causes acute hepatitis^1,2^. The virus is transmitted by the faeco-oral route or through close physical contact and can survive on surfaces, uncooked foods and in the environment for significant periods^3^. Hepatitis A infections are usually asymptomatic, though this is more common in early childhood. Symptomatic presentations could include nausea, vomiting, fatigue, or fever; symptoms that can easily be misattributed to the common cold, leading to underreporting of HAV infections^4^. Hence, 1.5 million clinical cases of hepatitis A occur worldwide annually, with an infection rate predicted to be ten times higher^5^. While HAV does not lead to chronic infections, it can lead to acute liver failure and other serious complications, accounting for 34,000 deaths yearly^6,7^.

In highly endemic areas, such as the Middle East and North Africa (MENA) region, more than 90% of the population would be expected to have natural immunity to the virus by the age of 10^5,7^. This high endemicity nullified the need for any large-scale immunization program in the region. However, the U.A.E., with its improved sanitation and socioeconomic status, has shown decreased seroprevalence and an increased average age of infection^8^. Hence, more cases occur in adulthood, where the burden and the risk of complications are higher^9,10^. Luckily, considerable reductions in morbidity and mortality could be achieved as a result of a powerful herd immunity effect^11^.

The mainstays of prevention encompass decreasing disease transmission, increasing population immunity and raising public awareness. Preventative measures such as vaccination and improved general knowledge are imperative to eradicate and prevent epidemics of communicable diseases such as hepatitis^12^. Given the limited treatment options for HAV infections, such measures are crucial to control and limit the spread of disease. In the U.A.E., HAV vaccination is not in the National Immunization Program unlike neighbouring countries such as Saudi Arabia, Bahrain, and Qatar^13^.

The few studies that evaluated the knowledge, attitudes and practices towards Hepatitis A worldwide reported low levels of knowledge, coupled with poor practices but positive attitudes^14–17^. This study aimed to assess the U.A.E. society’s hepatitis A knowledge, and attitudes and practices towards its vaccine, with the applicability of its introduction into the local immunization schedule.

## Methodology

### Study population

This cross-sectional, descriptive study was designed to collect comprehensive data from U.A.E. residents of the four most populous emirates: Abu Dhabi, Dubai, Sharjah and Ajman. These cities encompass the majority of the U.A.E.’s population and are the commercial and cultural hubs of the country. It was conducted between the months of January 2020 and March 2020 using convenience sampling from several public venues. 550 individuals were approached out of which 458 completed the questionnaire, yielding a response rate of 83%. Written informed consent was obtained from all participants. Visitors and tourists, those who cannot speak Arabic or English, and individuals under the age of 18 or with mental disabilities were excluded.

### Questionnaire development

Due to the lack of an existing tool for exploring HAV knowledge and vaccination practices in both English and Arabic, one was developed. The literature related to HAV, such as signs, symptoms, transmission and complications was reviewed^1,3,5,7,11,18,19^. Additionally, similar studies, either looking at HAV or other hepatitis viruses, were used for guidance on how to structure and grade the questionnaire^10,15–17,20^. The 50-item self-administered questionnaire consisted of different sections: demographics, HAV knowledge, immunization attitudes and practices, and sources of knowledge, in addition to their trustworthiness. It included 5-item Likert scales, true and false and multiple-choice questions.

The questionnaire was originally developed in English and then translated to Arabic. The Arabic version was reviewed multiple times to ensure consistency with the original. Language specialists and a biostatistician reviewed both versions. Both were pilot tested three times; all provided feedback was evaluated and incorporated if appropriate. Both, the English and Arabic questionnaires are included in Supplementary Information, items S1 and S2. This research was reviewed and approved by the Research Ethics Committee of the University of Sharjah (Reference Number: REC-20-01-29-01-S). It was conducted in accordance with all relevant guidelines and regulations.

### Study outcomes

This study explored the determinants of HAV knowledge and both attitudes and vaccination practices. Additionally, it evaluated the determinants of immunization schedule knowledge, attitudes, and practices. The second set of outcomes determined who would: know more about the schedule, receive the HAV vaccine themselves, and be more likely to trust the immunization schedule. Finally, it evaluated the effect of introducing the vaccine into the schedule on vaccination uptake rates.

### Data entry and analysis

The data was entered into IBM SPSS Statistics for Windows, Version 26.0 (IBM Corp., Armonk, NY, USA)^21^ and checked for any errors. Knowledge, attitudes and practices scores were generated for each participant. Additionally, each was asked questions regarding their perceived knowledge of HAV. For knowledge questions, a point was awarded for every correct answer; no points were deducted for skipped or incorrect answers. For attitudes and practices, positive points were awarded based on the grading of the responses, with more positive responses receiving more points. Five-item Likert scales were collapsed into three-item scales. Similar categories with very few participants were combined. Any missing values were labelled as such and were handled using pair-wise deletion.

For the univariate analysis, the data was exported in CSV format and visualised using the Matplotlib-v3.2.1 package^22^. First, normality of the continuous outcomes was evaluated using both Q–Q plots and Kolmogorov–Smirnov test. All reported percentages were calculated by excluding the missing values (valid percentages). All demographic variables, perceived knowledge and source of HAV information were evaluated as predictors for the outcomes. Bivariate analyses were conducted to identify significant predictors using Chi-square for non-continuous outcomes and Mann–Whitney U and Kruskal–Wallis H tests (supplemented with post-hoc Bonferroni corrections) for continuous outcomes. The Wilcoxon Signed Rank test was used to evaluate the matched difference. The cut-off for significance was a P value less than 0.05.

The data was then imported into R-4.0.0^23^ for multivariate analyses to evaluate the significance of the predictors collectively. All determinants were categorical and hence dummy coded. Initially, Ordinary Least Squares regression was used for the three continuous outcomes. However, heteroskedasticity was present (Studentized Breusch-Pagan tests gave a P < 0.0005 for all three models) and could not be adequately resolved through any transformations. Hence, Multiple Linear Regression with robust standard errors (HC1 estimator) was used. No outliers were detected. For each linear regression model, the minimum number of cases was met. They were calculated using $50+8m$, where $m$ is the number of predictors. For logistic regression, each model had more than 20 cases per predictor. Associations between determinants were tested for using Chi-square test of Independence. No interactions were explored. F score and R-squared values were calculated for each model.

For categorical and ordinal dependent variables, binary and ordinal logistic regression models were built. Both Proportional Odds Model (POM) and Partial Proportional Odds Model (PPOM) were used. These models can be parametrized in different ways. Different models’ coefficients are interpreted in different ways. Hence, the following parametrization was used where positive coefficients indicate higher odds for higher values of the dependent variable.

Assume an ordinal variable $Y$ with ordered categories $1, 2, 3, \dots , g, \dots , k$ and $p$ predictors, ${X}_{1},{X}_{2},\dots , {X}_{p}$. The POM would be:

1 $${\text{logit}}\left( {P\left( {Y \le g} \right)} \right) = \ln \frac{{P\left( {Y \le g} \right)}}{P(Y > g)} = \alpha_{g} - \beta_{1} X_{1} - \beta_{2} X_{2} \ldots - \beta_{p} X_{p} , \left( {g = 1, \ldots , k - 1} \right)$$

However, for a PPOM, the coefficients are allowed to vary by $g$:

2 $${\text{logit}}\left( {P\left( {Y \le g} \right)} \right) = \ln \frac{{P\left( {Y \le g} \right)}}{P(Y > g)} = \alpha_{g} - \beta_{1, g} X_{1} - \beta_{2, g} X_{2} \ldots - \beta_{p, g} X_{p} , \left( {g = 1, \ldots k - 1} \right)$$

POMs assume that odds-ratios are the same across cumulative splits of the data. To check for this assumption, the Brant test was used. When the assumption was violated, a multinomial logistic regression model was used to determine the offending variables. If so, PPOMs were used to estimate additional coefficients, as such models are able to relax the constraint of every variable having a common response for each predictor.

Adjusted odds-ratios were calculated using the estimated coefficients. To check for the model goodness of fit and overall significance, Hosmer–Lemeshow Test and the Likelihood Ratio Test were conducted, when applicable. The Hosmer–Lemeshow Test evaluates goodness-of-fit with the null hypothesis stating that the model is a good fit for the data. The Likelihood Ratio Test evaluates whether a model is better, either relative to a null or another specified model.

All P values reported are two-sided and all confidence intervals are profile confidence intervals.

## Results

### Demographics

Out of the 458 participants, 55% were females and 54% were married. Only 11% of our sample were Emiratis (U.A.E. nationals) which is expected as they constitute nearly 11% of the population^24^. More than 60% were university graduates. Finally, 7% were employed in the medical field. Table 1 represents the various proportions of different categories, along with the different knowledge, attitudes and practices outcomes.

**Table 1:** The table summarizes the most values collected using the questionnaire.

Demographics (Total N = 458)			Categorical outcomes (Total N = 458)				
	Categories	% (N)		Categories	% (N)		
Sex (Missing = 8)	Males	44.9% (n = 202)	Would you like to learn more about HAV? (Missing = 25)	Yes	84.3% (n = 365)		
	Females	55.1% (n = 248)		No	15.7% (n = 68)		
Age (Missing = 8)	18–29 years	44.2% (n = 199)	Would you get the HAV Vaccine? (Missing = 14)	Not planning to	23.6% (n = 105)		
	30–39 years	34.0% (n = 153)		Neutral	39.9% (n = 177)		
	40 + years	21.8% (n = 98)		Taken/planning to	36.5% (n = 162)		
Education (Missing = 20)	High School or Lower	26.3% (n = 115)	General source of knowledge (Missing = 24)	Physician	63.6% (n = 276)		
	Diploma	11.9% (n = 52)		Internet/social media	28.3% (n = 123)		
	Bachelor’s Degree	53.0% (n = 232)		Other	8.1% (n = 35)		
	Graduate Degree	8.9% (n = 39)	Vaccination source of knowledge (Missing = 24)	Physician	63.8% (n = 277)		
Nationality (Missing = 17)	Emirati	11.3% (n = 50)		Internet/social media	28.8% (n = 125)		
	Other Arab	49.4% (n = 218)		Other	7.4% (n = 32)		
	Non-Arab	39.2% (n = 173)	HAV Source of knowledge (Missing = 20)	Physician	53.9% (n = 236)		
Marital status (missing = 12)	Married	53.6% (n = 239)		Internet/social media	27.6% (n = 121)		
	Single or Otherwise	46.4% (n = 207)		Other	18.5% (n = 81)		
Residence (Missing = 2)	Abu Dhabi	26.9% (n = 123)	Barriers to Vaccination (Can select more than one) (Missing = 3)	Cost	39.6% (n = 180)		
	Ajman	22.1% (n = 101)		Availability	39.8% (n = 181)		
	Dubai	24.9% (n = 114)		Safety	48.6% (n = 221)		
	Sharjah and other Northern Emirates	25.7% (n = 118)		Unaware of benefits	46.2% (n = 210)		
			Immunization schedule knowledge (Missing = 7)	Identified correct vaccine	10.2% (n = 46)		
Occupation (Missing = 50)	Medical field	7.1% (n = 29)		Did not identify	89.8% (n = 405)		
	Unemployed	22.1% (n = 90)	Immunization schedule importance (missing = 10)	Not at all/slightly	9.4% (n = 42)		
	Non-medical field	70.8% (n-289)		Moderately	11.4% (n = 51)		
				Very/extremely	79.2% (n = 355)		

Continuous variables							
Variable	Mean	Median	Standard Deviation	Minimum	Maximum	Kolmogorov–Smirnov tests	
						Statistic	P value
Attitudes score	12	13	3.2	1	16	0.125	< 0.0005
Knowledge score	11.4	12	4.8	0	24	0.198	< 0.0005
Practices score	2.2	2	1.6	0	6	0.133	< 0.0005

It includes all determinants and outcomes explored in the paper. Kolmogorov–Smirnov tests were also supplemented with Q–Q plots. With P < 0.05, both showed a lack of normality for all three variables.

### Knowledge and knowledge sources

#### Univariate results

**Figure 1:**
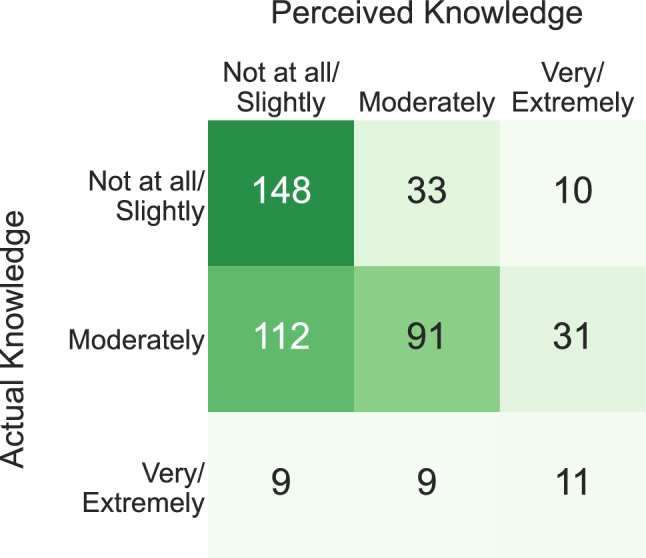
The different perceived and actual knowledge for the participants are cross-tabulated after collapsing the 5-item scales into 3-item scales.

In this study, each participant has both a perceived knowledge score and an actual knowledge score. Figure 1 represents the distribution of both perceived and actual knowledge for each respondent. Both were low with 42% of participants having poor knowledge and 59% stating such. Only 42% were able to correctly identify HAV transmission sources. Additionally, more than 30% incorrectly stated that HAV is a deportable disease. While 70% correctly identified that hepatitis A has a vaccine, more than 50% falsely presumed that all hepatitis viruses do. Only 10% were able to correctly identify hepatitis B vaccine as the only hepatitis vaccine in the National Immunization Schedule. 84% of the participants were interested in learning more about HAV.

**Figure 2:**
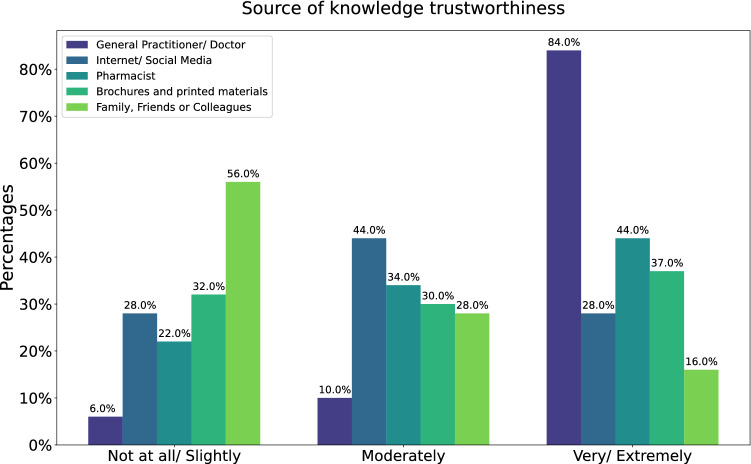
The figure shows how trustworthy different sources of knowledge were ranked by participants using 5-item Likert scales. During analysis, the responses were collapsed into 3-item scales.

Regarding the main sources of knowledge, 64% would consult a physician for any general medical or vaccine related concern but only 54% when looking for hepatitis A information. Additionally, internet and social media were the main source of knowledge for one out of every four participants. Moreover, Figure 2 demonstrates the trustworthiness of the different sources according to the participants. 84% ranked physicians as highly trustworthy while family, friends and colleagues were considered slightly trustworthy by more than half of the participants.

#### Bivariate and multivariate results

The outcomes of interest were knowledge score (a continuous variable) and local immunization schedule knowledge (a binary variable). The distributions of both are presented in Table 1.

Sex (P = 0.027), Occupation (P < 0.0005), Education (P = 0.001), and Perceived Knowledge (P < 0.0005) were found to be statistically significant predictors of knowledge score after bivariate analysis. Results of the multiple linear regression showed that all predictors remained significant except Sex, as displayed in Table 2. A Medical occupation (P = 0.009), a Bachelor’s degree (P = 0.011), and Moderate and High Perceived Knowledge (Both P values < 0.0005) were significant predictors for higher knowledge scores. The F-test showed that this model is a significant improvement over the intercept-only model.

**Table 2:** Multiple linear regression models - Knowledge and Attitudes.

Knowledge score–multiple regression (with robust standard errors)				
Model Terms	β-coefficient	RSE	t-Statistic	P value
Intercept (${\beta }_{0}$)	8.214	0.559	14.703	** < 0.0005**
**Sex (P = 0.027)**				
Male	–	–	–	**–**
Female	0.52	0.491	1.06	0.29
**Occupation (P = < 0.0005)**				
Non-medical	–	–	–	**–**
**Medical**	**2.069**	**0.791**	**2.616**	**0.009**
Unemployed	0.447	0.589	0.759	0.448
**Education (P = 0.001)**				
High school or lower	–	–	–	–
Diploma	0.852	0.733	1.161	0.246
**Bachelor’s**	**1.421**	**0.556**	**2.557**	**0.011**
Graduate	0.525	0.849	0.619	0.536
**Perceived knowledge (P < 0.0005)**				
Not at all/slightly	–	–	–	–
**Moderately**	**3.867**	**0.436**	**8.876**	** < 0.0005**
**Very/extremely**	**5.679**	**0.723**	**7.855**	** < 0.0005**
**R-squared: 25.21%**				
Adjusted R-squared: 23.61%	F (8, 374) = 19.05 **P < 0.0005**			

**Attitudes score–multiple regression (with robust standard errors)**				
Model terms	β-coefficient	RSE	t-Statistic	P value
Intercept (${\beta }_{0}$)	9.275	0.536	17.302	** < 0.0005**
**Sex (P = 0.008)**				
Male	–	–	–	–
**Female**	**0.653**	**0.31**	**2.107**	**0.036**
**Education (P = 0.001)**				
High school or lower	–	–	–	–
Diploma	0.277	0.612	0.453	0.651
**Bachelor’s**	**0.736**	**0.372**	**1.98**	**0.048**
**Graduate**	**1.187**	**0.496**	**2.392**	**0.017**
**Marital status (P = 0.001)**				
Single/other	–	–	–	–
**Married**	**0.639**	**0.302**	**2.113**	**0.035**
**Nationality (P = 0.033)**				
Non-Arab	–	–	–	–
Emirati	0.858	0.574	1.496	0.135
Other Arab	0.327	0.316	1.033	0.302
**Source for HAV information (P < 0.0005)**				
Other sources	–	–	–	–
**Physician**	**1.84**	**0.427**	**4.307**	** < 0.0005**
**Social media/internet**	**1.509**	**0.5**	**3.018**	**0.003**
**R-squared: 10.60%**				
Adjusted R-squared: 8.54%	F (9, 391) = 4.939 **P < 0.0005**			

The table represents the results of the knowledge and attitudes multiple linear regression models. P values that are significant at the 5% level are bolded. The significance level of the bivariate analyses is displayed under each predictor. This reflects the P value for the corresponding test with respect to the outcome variable (Mann–Whitney U for binary predictor variables, or Kruskal–Wallis for those with more than two groups). Robust Standard errors were used with an HC1 estimator. The knowledge model contains every significant predictor. For the attitudes model, age and actual knowledge were not included. Both predictors showed high multicollinearity with other features and hence were dropped.

*RSE* robust standard errors, *HAV* Hepatitis A Virus.

As for Immunization Schedule Knowledge, only Sex (P = 0.005) and Nationality (P = 0.019) were found to be significant. Both were fed into a binary logistic regression model, the results of which are shown in Table 3. Only Sex remained a significant predictor of better schedule knowledge. The odds of females recognizing the schedule are three times as large as those for males (P = 0.004; CI 1.482–6.678). The Hosmer–Lemeshow Test showed that the logistic regression model appears to fit the data well (P = 1.000) and the Likelihood Ratio Test indicated that the current model is a significant improvement over the null one (P = 0.006).

### Attitudes

#### Univariate analysis

Overall attitudes were positive. There was a near unanimous agreement that vaccination is important. Nearly 90% recognized that vaccines are available for the elderly with 68% agreeing that they should receive vaccines. Three out of every four participants found the local immunization schedule highly important when deciding to vaccinate their children. On the other hand, only 52% recognized that protection from vaccination decreases with age and 16% believed that vaccines cause autism. Finally, 40% of participants identified vaccine safety concerns, availability, cost, and a lack of knowledge regarding their benefits as significant barriers to vaccination.

#### Bivariate and multivariate analysis

The outcomes of interest were attitudes score (a continuous variable) and local immunization schedule importance (an ordinal variable). The distributions of both are presented in Table 1.

For the attitudes score, all the variables except Perceived Knowledge and Occupation were significant (P < 0.05). Hence, both multicollinearity and overfitting were of concern. The variables were assessed for any associations using Chi-Square tests. Actual Knowledge and Age were dropped due to high multicollinearity. The remaining variables were fed into a multiple linear regression model; results of which are presented in Table 2. Other than Nationality, all predictors remained significant. Those who were Female (P = 0.036), Married (P = 0.035) College educated (Bachelor’s: P = 0.048 and Graduate: P = 0.017), and whose main HAV source of knowledge was a Physician (P < 0.0005) or Social media/Internet(P = 0.003) had higher attitudes scores. The F-test showed that this model is a significant improvement over the intercept-only model.

As for the immunization schedule importance, bivariate analysis showed both Sex (P = 0.002) and Source of HAV Information (P = 0.037) as significant predictors. Both were fed into a proportional odds model (POM) and remained significant, presented in Table 3. When it came to ranking the immunization schedule as highly important, rather than a lower ranking, the odds of females were 2.141 times as large as those for males (P = 0.002; CI 1.310–3.499). Additionally, the odds for those whose main source of knowledge was a physician were 2.248 as large as those who would refer to other sources (non-Physician, non-Internet) (P = 0.007; CI 1.244–4.060). The Brant test showed that the parallel regression assumption holds (P = 0.47) and was confirmed by a multinomial logit model for odds ratio analysis. The Likelihood Ratio Test also indicated that the model is a significant improvement over the null model (P = 0.001).

### Practices

#### Univariate

Overall, participants’ practices were lacking. 81% of participants were vaccinated as a child, yet more than 75% were either unaware or did not take the hepatitis A and/or B vaccines. While 37% had taken or were planning to take the HAV vaccine, 40% needed more information before making a decision.

#### Bivariate and multivariate

**Figure 3:**
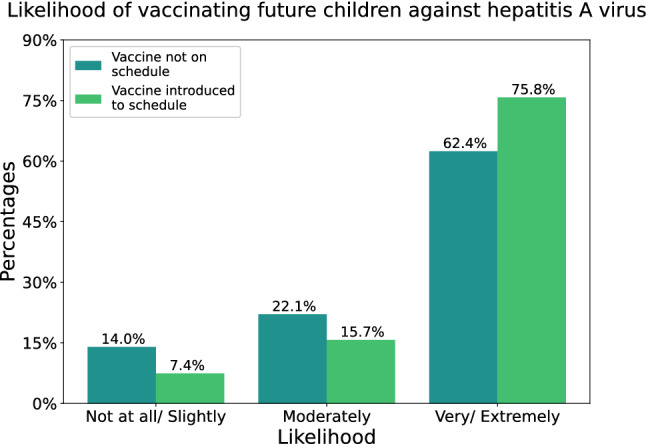
This figure shows the likelihood of participants vaccinating their future children against HAV, both before and after the vaccine is introduced into the schedule.

Respondents were asked whether they would vaccinate their future children against HAV, both before and after the vaccine is introduced into the National Immunization Program. As seen in Figure 3, those who are highly likely to vaccinate their children would increase more than 13% once the vaccine is introduced. A Wilcoxon Signed-Rank Test was used to evaluate whether the increase was significant. The P value for the test was < 0.0005 indicating better vaccine uptake among the community post-vaccine introduction.

As with knowledge and attitudes, the determinants of both the practices score (a continuous variable) and Willingness to Get Vaccinated (an ordinal variable) were analysed using bivariate techniques.

For the practices score, Sex (P < 0.0005), Age (P = 0.013), Occupation (P = 0.001), Actual Knowledge (P < 0.0005), and Perceived Knowledge (P < 0.0005) were found to be statistically significant determinants. The variables were fed into a multiple linear regression model with the results presented in Table 4. All predictors remained significant except for occupation. Being Female (P < 0.0005), in the age groups of 30–39 years (P = 0.002) or 40 + years (P = 0.030), having moderate Actual Knowledge (P = 0.014), and high Perceived Knowledge (P = 0.011) were associated with better practices. The F-test showed that this model is a significant improvement over the intercept-only model.

**Table 3:** Binary and ordinal logistic regression models.

Immunization schedule knowledge–binary logistic regression (LR)						
Model terms	${{\varvec{e}}}^{{{\varvec{\beta}}}_{{\varvec{i}}}}$	95% CI	SE	z-Statistic	P value	
Intercept (${\beta }_{0}$)	**0.045**	**0.020**–**0.088**	**0.374**	**− 8.305**	** < 0.0005**	
Sex (P = 0.005)						
Male	–	–	–	–	–	
**Female**	**3.019**	**1.482**–**6.678**	**0.380**	**2.906**	**0.004**	
Nationality (P = 0.019)						
Arab	–	–	–	–	–	
Non-Arab	1.513	0.778–2.911	0. 334	1.239	0.215	
Hosmer–Lemeshow test	Likelihood ratio test					
${\chi }^{2}$= 0.302	df = 8	P = 1.000	${{\varvec{\chi}}}^{2}$= **10.344**	**df = 2**	**P = 0.006**	

Immunization schedule importance—cumulative logit model with proportional odds (PPO)						
Coefficients	${{\varvec{e}}}^{{{\varvec{\beta}}}_{{\varvec{i}}}}$	95% CI	SE	z-Statistic	P value	
Sex (P = 0.002)						
Male	–	–	–	–	–	
**Female**	**2.141**	**1.310–3.499**	**0.251**	**3.037**	**0.002**	
Source for HAV information (P = 0.037)						
Others	–	–	–	–	–	
**Physician**	**2.248**	**1.244–4.060**	**0.302**	**2.685**	**0.007**	
Social media/internet	1.934	0.992–3.771	0.341	1.936	0.053	

Intercepts	${{\varvec{\beta}}}_{{\varvec{i}}}$	95% CI	SE	z-Statistic	P value	
Not at all/slightly|moderately	− 1.382	− 1.938–− 0.826	0.284	− 4.872	**< 0.0005**	
Moderately|very/extremely	− 0.443	− 0.963–0.077	0.265	− 1.671	0.095	
Brant test (parallel regression assumption)	Likelihood ratio test					
Omnibus ${\chi }^{2}$ = 2.55	df = 3	P = 0.47	${{\varvec{\chi}}}^{2}$= **17.484**	**df = 3**	**P = 0.001**	

Willingness to get vaccinated–cumulative logit model with partial proportional odds (PPOM)						
Coefficients	Outcome	${{\varvec{e}}}^{{{\varvec{\beta}}}_{{\varvec{i}}}}$	95% CI	SE	z-Statistic	P value
Age (P = 0.001)						
18–29 years	Will not take	–	–	–	–	–
30–39 years	Neutral	1.085	0.633–1.861	0.275	0.298	0.766
	**Taken or planning**	**1.740**	**1.085–2.789**	**0.241**	**2.300**	**0.021**
40 + years	Neutral	0.689	0.389–1.222	0.292	− 1.274	0.203
	Taken or planning	1.576	0.923–2.690	0.273	1.665	0.096
Perceived knowledge (P = 0.002)						
Not at all/few	Will not take	–	–	–	–	–
Moderately	Neutral	1.170	0.672–2.038	0.283	0.556	0.578
	**Taken or planning**	**2.135**	**1.333–3.421**	**0.241**	**3.153**	**0.002**
Very/extremely	Neutral	0.756	0.371–1.540	0.363	− 0.769	0.442
	**Taken or planning**	**1.988**	**1.032–3.828**	**0.334**	**2.055**	**0.040**
Actual knowledge (P < 0.0005)						
Not at all/few	Will not take	–	–	–	–	–
Moderately	**Both**	**1.620**	**1.097–2.393**	**0.199**	**2.423**	**0.015**
Very/extremely	Both	0.625	0.286–1.367	0.399	− 1.176	0.240

Intercepts	${{\varvec{\beta}}}_{0, {\varvec{i}}}$	95% CI	SE	z-Statistic	P value	
Will not take | Neutral	− 1.054	− 1.456–0.652	0.205	− 5.137	**< 0.0005**	
Neutral | Taken or Planning	1.414	1.010–1.818	0.206	6.856	**< 0.0005**	
Likelihood ratio test (relative to a null model)	Likelihood ratio test (relative to a proportional odds model)					
${{\varvec{\chi}}}^{2}$= **48.009**	**df = 10**	**P < 0.0005**	${{\varvec{\chi}}}^{2}$= **23.296**	**df = 4**	**P < 0.0005**	

The table represents the results of the logistic and ordinal regression models. The lower and upper bounds of the confidence interval were exponentiated for easier interpretation.

*CI* Confidence Interval, *SE* standard error, *HAV* Hepatitis A Virus.

Bivariate analysis indicated that Age (P = 0.001), Perceived knowledge (P = 0.002) and Actual knowledge (P < 0.0005) were significant determinants for the Willingness to Get Vaccinated. A POM was initially built but Brant test yielded a P < 0.0005. Further by-variable analysis revealed that Age and Perceived knowledge had different odds ratios across the 3 outcome levels. This was confirmed using a multinomial logit model. Proportional odds assumption was relaxed for both predictors. All three remained significant.

Given the nature of PPOM, multiple coefficients were generated, all shown in Table 3. When it came to receiving the vaccine as opposed to being neutral or not taking it, the odds of those who are 30–39 years old were 1.740 times as large as the younger population (P = 0.021; CI 1.085–2.789). As for perceived knowledge, both having moderate (AOR = 2.135; P = 0.002; CI 1.333–3.421) and high (AOR = 1.988; P = 0.040; CI 1.032–3.828) knowledge perception were associated with higher odds of taking the vaccine. Finally, the odds of those with moderate knowledge were 1.620 times larger to receive the vaccine than those with low levels of actual knowledge (P = 0.015; CI 1.097–2.393). Our model was tested against both a null model and the initial POM model. Both likelihood ratio tests indicated significant improvement over both (P < 0.0005) justifying the use for a PPOM.

**Table 4:** Multiple linear regression models - Practices.

**Practices score–multiple regression (with robust standard errors)**				
Model terms	β-coefficient	RSE	t-Statistic	P value
Intercept (${\beta }_{0}$)	1.069	0.141	7.571	** < 0.0005**
**Sex (P < 0.0005)**				
Male	–	–	–	–
**Female**	**0.669**	**0.172**	**3.881**	** < 0.0005**
**Age (P = 0.013)**				
18–29 years	–	–	–	–
**30–39 years**	**0.559**	**0.178**	**3.14**	**0.002**
**40 + years**	**0.46**	**0.212**	**2.174**	**0.03**
**Occupation (P = 0.001)**				
Non-medical field	–	–	–	–
Medical field	0.463	0.34	1.364	0.174
Unemployed	− 0.203	0.205	− 0.992	0.322
**Actual knowledge (P < 0.0005)**				
Not at all/slightly	–	–	–	–
**Moderately**	**0.426**	**0.172**	**2.477**	**0.014**
Very/extremely	0.188	0.222	0.849	0.397
Perceived knowledge **(P < 0.0005)**				
Not at all/slightly	–	–	–	–
Moderately	0.34	0.184	1.852	0.065
**Very/extremely**	**0.815**	**0.319**	**2.552**	**0.011**
**R-squared: 16.01%**				
Adjusted R-squared: 14.02%	F (9, 380) = 8.05 **P < 0.0005**			

The table represents the results of the practices multiple linear regression models. P values that are significant at the 5% level are bolded. The significance level of the bivariate analyses is displayed under each predictor. This reflects the P value for the corresponding test with respect to the outcome variable (Mann–Whitney U for binary predictor variables, or Kruskal–Wallis for those with more than two groups). Robust Standard errors were used with an HC1 estimator. The model contains every significant predictor.

*RSE* robust standard errors, *HAV* Hepatitis A Virus.

## Discussion

### Summary of results

This study aimed to assess the U.A.E. society’s knowledge, attitudes and practices to hepatitis A and its vaccine and the applicability of vaccine introduction into the National Immunization Program.

Overall, more than half of the participants had poor disease and vaccine knowledge. However, an overwhelming majority recognized this and were interested in learning more about HAV. In addition, participants showed great trust in physicians with more than half referring to them as their main source of knowledge and a higher proportion ranking them as highly trustworthy. Practices were also poor with three-fourths of the participants being unaware of their vaccination status for hepatitis A and/or B, despite more than 80% having received some childhood vaccinations. Additionally, more than a third stated that they would need more information before taking the HAV vaccine. Moreover, participants identified vaccine safety concerns, availability, costs, and a lack of knowledge regarding their benefits as significant barriers to vaccination.

Yet, attitudes were positive with a near unanimous agreement that vaccination is important. Moreover, most recognized that adult and elderly vaccination is possible but not as many appreciated their necessity. Participants additionally showed a high level of trust in the National Immunization Program with 80% ranking it as highly important. Furthermore, it positively influenced the participants with more people likely to vaccinate their children if the HAV vaccine was in the schedule. However, some negative attitudes were detected; only half recognized the waning of immunization protection with age and nearly one in six falsely believed that vaccination causes autism.

Multivariate analyses showed that being female was the strongest determinant of National Immunization Schedule knowledge and vaccination practices and attitudes. Knowledge perception was the second most significant factor, increasing with actual better knowledge and practices, as well as increased willingness to get vaccinated. Surprisingly, moderate knowledge, as opposed to high, was a significant factor for better vaccination practices. Moreover, having a university education and consulting a physician were associated with overall better vaccination attitudes. However, ironically, being a healthcare professional was only associated with higher hepatitis A knowledge, but not attitudes and practices.

### Validity

Given the convenience sampling used, the participants may not be representative of the U.A.E.’s overall population and the results may not be fully generalizable. However, care was taken during sampling to be inclusive and target a variety of different public settings in each city. Additionally, self-reported immunization attitudes and practices may not reflect actual behaviour. Vaccination status was self-reported by the participants and was not cross-referenced with medical records. Finally, no information regarding socioeconomic status (or an equivalent proxy) was collected.

### Comparison with existing literature

Very few studies have looked at HAV, both globally and in the MENA region. Even then, those that have explored it in the community found low levels of knowledge, coupled with poor practices^14–17^. In fact, this has also been documented among physicians. A U.S. study found a lack of strong and consistent recommendation of the HAV vaccine among both paediatricians and family medicine physicians, leading to suboptimal population coverage^10^. Given the limited number of HAV studies, results were compared with others investigating hepatitis B and hepatitis C in the region, all of which presented an equally gloomy picture. The studies found a general lack of knowledge regarding hepatitis viruses, coupled with confusion regarding the different viruses and overall poor practices^25–27^. As for knowledge sources, one study reported that only 26% of participants would consult a physician as first line^25^.

Even fewer studies looked at HAV vaccine and its attitudes. 75% of parents in a U.S. study agreed that the vaccine is safe for their children^16^. As for general vaccination attitudes, a study among Swedish parents reported that 79% unequivocally accepted any recommended vaccines^28^. Similarly, a U.A.E. study among parents showed positive vaccination attitudes with 90% agreeing that vaccines are important. However, the same study reported some negative attitudes, with a third of the parents reporting concerns regarding the side effects of vaccines^29^.

As for vaccine uptake determinants, a study among females found that positive attitudes were the most important in determining a child’s vaccination status^30^. Physicians have also been shown to be influential in the decision to vaccinate^10,16,31,32^. A systematic review found that eight out of nine studies showed that vaccine recommendation by a healthcare professional increased the uptake^33^. As for education level, there were mixed reviews, with some finding that higher education level was associated with less vaccination uptake^34^, and others showing the opposite^16^.

### Significance of results

This study shows that the community in the U.A.E. has low levels of knowledge regarding HAV and poor practices but highly positive attitudes, especially towards immunization. Most participants were willing to vaccinate against HAV if the vaccine was introduced into the National Immunization Schedule. The vaccine is both highly immunogenic and safe, generating long-lasting immunity without any safety concerns^35,36^. It affords long term HAV protection; antibodies persisting for at least twenty and possibly up to forty years and beyond^37–39^. In fact, current evidence does not recommend booster shots for fully vaccinated adults, highlighting the efficacy of the vaccine and demonstrating the role of childhood vaccination in protecting adults^40^. Therefore, the introduction of the vaccine in the U.A.E. guarantees protection against HAV into adulthood, potentially reducing the incidence and burden of clinical hepatitis A.

Globally, vaccination has been coming under fire as vaccine hesitancy, the act of delaying or refusing a subset of vaccines despite their availability, has taken root^41^. Its determinants are complex and multidimensional, encompassing a variety of social, political, religious, medical and economic factors^42^. Among them, vaccination information distrust has been shown to consistently lead to doubt in public health policies globally, including the immunization schedules^43^. Yet, the WHO’s Strategic Advisory Group on Experts highlights the importance of the schedules with regards to vaccine uptake and childhood immunization^41^. However, the results of this study reflect a deep trust in the U.A.E.’s National Immunization Program. Therefore, any national campaigns, in order to maintain and preserve the current trust, could take a highly proactive role, openly discussing and alleviating any vaccine hesitancy concerns before they spread in the community.

Even more alarming is the fact that healthcare professionals, vaccination’s main proponents, have been documented to exhibit vaccine hesitancy^44^. While no results are available for the U.A.E., this study’s participants highly ranked physicians, and a large majority considered them their main source of information for all health enquiries. Hence, it is vital that physicians are well-informed regarding vaccination and are encouraged to recommend the HAV vaccine. Additional studies would need to be conducted to try to ascertain any vaccine hesitancy among U.A.E. physicians.

While increased knowledge would be expected to correlate with reduced hesitancy, the relationship is far more complex. Some studies found that awareness and knowledge are predictors of higher vaccine acceptance among healthcare professionals only^45^. Even so, others highlighted that knowledge in the community still plays a role in vaccine uptake, but possibly overall immunization schedule knowledge as opposed to the individual vaccines^15,16,33^. However, there is strong evidence linking positive attitudes to vaccine uptake^33^. In fact, worries about vaccine side effects was the most common reason for not vaccinating a child^33^. This may hinder future immunization initiatives as vaccine safety concerns have been consistently shown to impair vaccine uptake^16,32,33,42^.

Internet and social media were not as highly ranked as other sources of information; however, participants still heavily relied on them. In fact, a study among mothers found that such Web 2.0 technologies were becoming vital and trusted information sources regarding vaccination^28,46^. While they can help promote and increase vaccine uptake rates, misinformation and anti-vaccination rhetoric thrives among the different platforms and their effect on parental decisions are yet to be determined^33,47^. Hence, such sources, while unable to push the community towards outright opposition, may sow the seeds of hesitancy. In Denmark, human papilloma virus vaccine uptake rate dropped from 90 to 54% after increased negative media coverage^48^. A study among Chinese parents found that referring to the internet was associated with increased concerns for vaccination^49^. Hence, monitoring and promoting vaccination among Web 2.0 technologies may be essential to maintain the trust in the National Immunization Program.

## Conclusion

This study found poor knowledge and practices in the U.A.E. coupled with positive attitudes towards HAV and its vaccine. Participants nearly unanimously agreed that vaccination is important. National campaigns could maintain this trust by alleviating any vaccine hesitancy concerns before they spread in the community. Such concerns have been consistently shown to impair vaccine uptake rates. The majority of participants showed a high level of trust in the National Immunization Program. More were likely to vaccinate their children if the HAV vaccine was introduced into the schedule. The vaccine would guarantee protection against HAV into adulthood, potentially reducing the incidence and burden of clinical hepatitis A in the U.A.E.

For sources of information, participants showed great trust in physicians with many finding them to be highly trustworthy. Hence, physicians need to be well-informed regarding vaccination and encouraged to recommend the HAV vaccine. However, participants still relied heavily on the internet and social media as other sources of information. Hence, monitoring and promoting vaccination among those technologies may be essential to maintain the trust in the National Immunization Program.

## Supplementary Information

Supplementary Information

## Data Availability

All data supporting the findings of this study are available within the article and the Supplementary Information file, or are available from the corresponding authors on reasonable request.
