# Supplementary material for: Hepatitis A virus knowledge and immunization attitudes and practices in the United Arab Emirates community
Source: Sci Rep. 2021 Jan 29;11:2651. doi: 10.1038/s41598-020-80089-4 (PMC7846788; doi:10.1038/s41598-020-80089-4)
Supplement: Supplementary file 1 — Supplementary Information [file 41598_2020_80089_MOESM1_ESM.pdf]

# Hepatitis A Virus knowledge and immunization attitudes and practices in the United Arab Emirates community

*Kamel A. Samara[1,*], Hiba J. Barqawi[2,**], Basant H. Aboelsoud[1], Moza A. Alzaabi[1], Fay T. Alraddawi[1], Ayten A. Manna[1]*

[1]College of Medicine, University of Sharjah, Sharjah, United Arab Emirates

[2]Department of Clinical Sciences, College of Medicine, University of Sharjah, United Arab Emirates

[*]Corresponding author. Email: kamel.samara@hotmail.com

[**]Corresponding author. Email: hbarqawi@sharjah.ac.ae

## Supplementary Information

S1 is the English questionnaire used for data collection.

S2 is the Arabic questionnaire used for data collection.

UNIVERSITY OF SHARJAH
where civilizations meet

# Hepatitis A Virus knowledge and immunization attitudes and practices in the United Arab Emirates community

## Demographic Data

***Please circle the option that best describes you***

| | | | | |
|---|---|---|---|---|
| **1) Sex** | 1) Male | | 2) Female | |
| **2) Age** | 1) 18-29 | 2) 30-39 | 3) 40-49 | 4) 50 and above |

| | | |
|---|---|---|
| **3) Highest degree obtained** | 1) Middle School or lower | 2) High School |
| 3) Diploma | 4) University Degree | 5) Graduate Degree (MSc, PhD., etc.) |

| | | | |
|---|---|---|---|
| **4) Marital Status** | 1) Single | 2) Married | 3) Other (____________) |
| **5) Nationality** | 1) Emirati | 2) Other Arab | 3) Non-Arab |
| **6) Occupation** | 1) Unemployed | 2) Medical Field | 3) Business/Finance |
| 4) Government/Law | 5) Education | 6) Arts and Communication | |
| 7) Administration, Engineering or I.T. | 8) Student (Major - ______________) | | |
| **7) Place of Residence** | 1) Sharjah | 2) Dubai | 3) Fujairah |
| 4) Umm Al Quwain | 5) Ajman | 6) Abu Dhabi | 7) Ras Al Khaimah |

## Knowledge, Attitudes and Practices about Hepatitis Virus Infections:

**8) Have you heard of 'Hepatitis Virus'?**

☐ 1) Yes ☐ 2) No (Go to Question 24)

**9) How knowledgeable are you about Hepatitis Viruses?**

| | | | | |
|---|---|---|---|---|
| 1<br>Not at all | 2<br>Slightly | 3<br>Moderately | 4<br>Very | 5<br>Extremely |

**10) How knowledgeable are you about Hepatitis A Virus?**

| | | | | |
|---|---|---|---|---|
| 1<br>Not at all (Go to Question 24) | 2<br>Slightly | 3<br>Moderately | 4<br>Very | 5<br>Extremely |

**11) Which of the following are hepatitis viruses that can infect humans? (*You can select more than one option*)?**

- ☐ 1) Hepatitis A
- ☐ 2) Hepatitis B
- ☐ 3) Hepatitis C
- ☐ 4) Hepatitis D
- ☐ 5) Hepatitis E
- ☐ 6) Hepatitis F
- ☐ 7) None of the above

**12) How prevalent do you think Hepatitis A infections are?**

| 1<br>Not at all | 2<br>Slightly | 3<br>Moderately | 4<br>Very | 5<br>Extremely |
|---|---|---|---|---|

**13) How fatal do you think Hepatitis A infections are?**

| 1<br>Not at all | 2<br>Slightly | 3<br>Moderately | 4<br>Very | 5<br>Extremely |
|---|---|---|---|---|

**State whether the following statements about HEPATITIS A are 'True' or 'False'.**

| Statement | 1) True | 2) False | 3) Don't know |
|---|---|---|---|
| 14) Infections are long-term | | | |
| 15) Most infections do not show symptoms in children | | | |
| 16) The older the patient, the less serious the infection. | | | |
| 17) Infection causes sickness within 48 hours. | | | |
| 18) There are medications that can cure the infection. | | | |
| 19) In some cases, the infection can lead to liver failure. | | | |
| 20) It is screened for during the medical fitness test in the United Arab Emirates. | | | |
| 21) Exposure protects against future infection. | | | |

**22) Hepatitis A can be transmitted through which of the following?** (You can select more than one option)

- ☐ 1) Contaminated Food or Water.
- ☐ 2) Direct Contact with Hepatitis A patients.
- ☐ 3) Sharing needles or blood exchange.
- ☐ 4) Sexual intercourse with infected patients.
- ☐ 5) Air & Sneezing.
- ☐ 6) Sharing clothes and towels.

**23) Hepatitis A infections can cause which of the following?** (You can select more than one option)

- ☐ 1) Yellow skin or eyes
- ☐ 2) Vomiting
- ☐ 3) Stomach Pain
- ☐ 4) Feeling tired
- ☐ 5) Fever
- ☐ 6) Joint Pain

**24) A deportable disease is a one that would cause a person to be removed forcibly from the country. Which of the following do you think is deportable?** (You can select more than one option)

☐ 1) HIV ☐ 2) Cancer ☐ 3) Hepatitis A
☐ 4) Hepatitis B ☐ 5) Hepatitis C ☐ 6) Syphilis
☐ 7) Tuberculosis ☐ 8) Mad Cow Disease ☐ 9) Malaria
☐ 10) Leprosy ☐ 11) Chlamydia ☐ 12) HPV

## Hepatitis viruses & Vaccination

**Do you agree or disagree with the following statements?**

| Statement | 1) Agree | 2) Disagree |
|---|---|---|
| 25) Vaccines are important to protect children from diseases. | | |
| 26) Vaccines are available for adults and elderly. | | |
| 27) All Hepatitis viruses have vaccines. | | |
| 28) Vaccination causes autism. | | |
| 29) It is not necessary that adults and elderly get vaccinated. | | |
| 30) Hepatitis A Virus has a vaccine. | | |
| 31) Protection from vaccination decreases with age. | | |

**32) Were you vaccinated as a child from any disease?**

☐ 1) Yes ☐ 2) No *(Go to 35)* ☐ 3) I don't remember *(Go to 35)*

**33) Were you vaccinated against Hepatitis A?**

☐ 1) Yes ☐ 2) No ☐ 3) I don't remember

**34) Were you vaccinated against Hepatitis B?**

☐ 1) Yes ☐ 2) No ☐ 3) I don't remember

**35) Have you received any vaccines as an adult?**

☐ 1) Yes, Specify (__________ ____________________________) ☐ 2) No

**36) Have you received the Hepatitis A vaccine as an adult?**

☐ 1) Yes, I've taken it ☐ 2) No and I don't plan on taking it

☐ 3) No, but I'm planning on taking it ☐ 4) I need more information to decide

**37) Do you know which Hepatitis Viruses are part of the U.A.E.'s immunization schedule *(You can choose more than one)?***

☐ 1) Hepatitis A ☐ 2) Hepatitis B ☐ 3) Hepatitis C
☐ 4) Hepatitis D ☐ 5) Hepatitis E ☐ 6) Hepatitis F
☐ 7) I don't know

**38) How important is the U.A.E.'s immunization schedule to you when vaccinating your children?**

| 1<br>Not at all | 2<br>Slightly | 3<br>Moderately | 4<br>Very | 5<br>Extremely |
|---|---|---|---|---|

**39) How likely are you to vaccinate your future children against Hepatitis A, keeping in mind that Hepatitis A Virus vaccine is not part of the U.A.E.'s immunization schedule?**

| 1<br>Not at all | 2<br>Slightly | 3<br>Moderately | 4<br>Very | 5<br>Extremely |
|---|---|---|---|---|

**40) If the vaccine were to be added to the immunization schedule, how likely are you to vaccinate your future children against Hepatitis A?**

| 1<br>Not at all | 2<br>Slightly | 3<br>Moderately | 4<br>Very | 5<br>Extremely |
|---|---|---|---|---|

**41) What do you think is the BIGGEST barrier to vaccination? (Choose all that apply)**

☐ 1) Cost ☐ 2) Availability ☐ 3) Concerns about safety
☐ 4) Unaware of benefits ☐ 5) Others, specify (______________)

## Knowledge Sources and Credibility

**42) If you wanted to learn more about a disease or medical condition, which of the following would be your MAIN source of knowledge? *(Choose one only)***

☐ 1) General Practitioner/ Doctor ☐ 2) Internet/Social Media ☐ 3) Pharmacist
☐ 4) Brochures & other printed materials ☐ 5) Family, Friends or Colleagues ☐ 6) Others, specify (______________)

**43) If you wanted to learn more about a vaccine or its side effects, which of the following is your MAIN source of knowledge? *(Choose one only)***

- ☐ 1) General Practitioner/ Doctor
- ☐ 2) Internet/Social Media
- ☐ 3) Pharmacist
- ☐ 4) Brochures & other printed materials
- ☐ 5) Family, Friends or Colleagues
- ☐ 6) Others, specify (______________)

**44) Would you be interested in learning more about Hepatitis A?**

- ☐ 1) Yes
- ☐ 2) No (Go to Question 46)

**45) To learn more about Hepatitis A, which of the following would be your MAIN source of knowledge? *(Choose one only)***

- ☐ 1) General Practitioner/Doctor
- ☐ 2) Internet/Social Media
- ☐ 3) Pharmacist
- ☐ 4) Brochures & other printed materials
- ☐ 5) Family, Friends or Colleagues
- ☐ 6) Others, specify (_____________)

**How trustworthy is each of the following sources?**

| **Source** | **Level of Trust** | | | | |
|---|---|---|---|---|---|
| | **1)Not at all** | **2)Slightly** | **3)Moderately** | **4)Very** | **5)Extremely** |
| 46) General Practitioner/Doctor | | | | | |
| 47) Internet/ Social Media | | | | | |
| 48) Pharmacist | | | | | |
| 49) Brochures and printed materials | | | | | |
| 50) Family, Friends or Colleagues | | | | | |

***Thank you for your participation!***

UNIVERSITY OF SHARJAH
where civilizations meet

# السلوكيات والممارسات المعرفية لفيروس التهاب الكبد الوبائي (أ) والتحصين في مجتمع الإمارات العربية المتحدة

## البيانات الديموغرافية - اختر الإجابة التي تناسبك

| | | | | |
|---|---|---|---|---|
| 1) الجنس | 1) ذكر | | 2) أنثى | |
| 2) العمر | 1) 18-29 | 2) 30-39 | 3) 40-49 | 4) 50 فما فوق |
| 3) أعلى شهادة حاصل عليها | 1) مدرسة إعدادية أو أقل | 2) مدرسة ثانوية | 3) دبلوم مهني | 4) شهادة جامعية | 5) دراسات عليا |
| 4) الحالة الاجتماعية | 1) أعزب | 2) متزوج | 3) غير ذلك (__________) | |
| 5) الجنسية | 1) إماراتي | 2) جنسية عربية أخرى | 3) أجنبي | |
| 6) الجهة الوظيفية | 1) غير موظف | 2) المجال الطبي | 3) الجهة المالية / محاسبة | 4) مجال القانون |
| 5) القطاع التعليمي | 6) المجال الفني / علاقات عامة | 7)الإدارة/ الهندسة / علوم الحاسوب | 8) طالب (التخصص - ________________) | |
| 7) مكان الإقامة | 1) الشارقة | 2) دبي | 3) الفجيرة | |
| 4) أم القيوين | 5) عجمان | 6) أبوظبي | 7) رأس الخيمة | |

## السلوكيات المعرفية تجاه التهاب الكبد الوبائي – A

**8) هل سمعت عن فيروس التهاب الكبد ؟**

☐ **1- نعم** ☐ **2- لا ( انتقل لسؤال 24 )**

**9) عبر عن مدى معرفتك لفيروسات التهاب الكبد ؟**

| | | | | |
|---|---|---|---|---|
| 1) أبدا | 2) قليلة | 3) متوسطة | 4) كبيرة | 5) كبيرة جدا |

**10) عبر عن مدى معرفتك لفيروسات التهاب الكبد A ؟**

| | | | | |
|---|---|---|---|---|
| 1) أبدا (انتقل لسؤال 24) | 2) قليلة | 3) متوسطة | 4) كبيرة | 5) كبيرة جدا |

**11) أي أنواع فيروسات التهاب الكبد يمكن أن تصيب الانسان؟ ( يمكن اختيار أكثر من إجابة )**

☐ 1) التهاب الكبد A
☐ 2) التهاب الكبد B
☐ 3) التهاب الكبد C
☐ 4) التهاب الكبد D
☐ 5) التهاب الكبد E
☐ 6) التهاب الكبد F
☐ 7) لا شيء مما ذكر

**12) ما مدى انتشار فيروس التهاب الكبد A ؟**

| | | | | |
|---|---|---|---|---|
| 1) أبدا | 2) قليلة | 3) متوسطة | 4) كبيرة | 5) كبيرة جدا |

**13) هل يعتبر فيروس التهاب الكبد A فتّاكاً ؟**

| | | | | |
|---|---|---|---|---|
| 1) أبدا | 2) قليلة | 3) متوسطة | 4) كبيرة | 5) كبيرة جدا |

**هل توافق على العبارات التالية المتعلقة بفيروس التهاب الكبد A أم لا؟**

| العبارة | 1)أوافق | 2)لا أوافق | 3) لا أعلم |
|---|---|---|---|
| 14) الأمراض المتعلقة به مزمنة | | | |
| 15) معظم أمراض التهاب الكبد **A** ليس لها أعراض على الأطفال | | | |
| 16) كلما كبر الشخص في العمر ، قلّت حدية المرض وأصبح فتاكاً بشكل أقل | | | |
| 17) العدوى تسبب المرض خلال **48** ساعة | | | |
| 18) يتوفر هناك أدوية لعلاج هذه العدوى | | | |
| 19) بعض الحالات (حال تطورها) يمكن أن تؤدي إلى فشل الكبد | | | |
| 20) يتم فحص الشخص الخاضع لاختبار اللياقة البدنية في دولة الإمارات / لأغراض تجديد الإقامة | | | |
| 21) التعرض للفيروس يقي من الإصابة به في المستقبل | | | |

**22) كيف يمكن لفيروس التهاب الكبد A أن ينتقل ؟ ( يمكن اختيار أكثر من إجابة )**

☐ 1) أطعمة / مياه ملوثة
☐ 2) اتصال مباشر مع شخص مصاب بفيروس التهاب الكبد A
☐ 3) المشاركة بالإبر / نقل الدم
☐ 4) اتصال جنسي بشخص مصاب بفيروس الكبد A
☐ 5) الهواء& العطاس
☐ 6) المشاركة بالمناشف /الملابس

**23) ما الذي يمكن أن يسببه التهاب الكبد A؟ ( يمكن اختيار أكثر من إجابة )**

☐ 1) اصفرار الجلد/ العينين
☐ 2) القيء
☐ 3) آلام المعدة

☐ 4) الشعور بالتعب ☐ 5) الحرارة ☐ 6) آلام المفاصل

**24) الأمراض الترحيلية، هي الأمراض التي تؤدي إلى ترحيل الشخص إجباريا إذا تم تأكيد حمله لها. ما الأمراض من الأمراض الأتية قد تظن أنها قد تؤدي للترحيل؟ ( يمكن اختيار أكثر من إجابة )**

☐ 1) فيروس نقص المناعة ☐ 2) السرطان ☐ 3) التهاب الكبد A

☐ 4) التهاب الكبد B ☐ 5) التهاب الكبد C ☐ 6) مرض الزهري ( السفلس )

☐ 7) مرض السل ☐ 8) مرض جنون البقر ☐ 9) الملاريا

☐ 10) الجذام ☐ 11) الكلاميديا ☐ 12) فيروس الورم الحليمي البشري

## فيروسات التهاب الكبد و التطعيم

**هل توافق أو لا توافق العبارات الأتية؟**

| العبارة | 1) أوافق | 2) لا أوافق |
|---|---|---|
| **25) التطعيم مهم جدا لحماية الأطفال من الأمراض.** | | |
| **26) التطعيم متوفر أيضا للبالغين و كبار السن.** | | |
| **27) جميع فيروسات التهاب الكبد لديها تلقيحات .** | | |
| **28) التطعيمات تسبب التوحد.** | | |
| **29) ليس من الضروري للبالغين و كبار السن أخذ التطعيمات.** | | |
| **30) فيروس التهاب الكبد A له تطعيم.** | | |
| **31) الحماية المكتسبة من التطعيم تتناقص مع تقدم العمر.** | | |

**32) هل تم تطعيمك ضد أي مرض عندما كنت صغيرا؟**

☐ **1- نعم** ☐ **2- لا (انتقل لسؤال 35)** ☐ **3- لا أتذكر (انتقل لسؤال 35)**

**33) هل تم تطعيمك ضد فيروس التهاب الكبد A؟**

☐ **1- نعم** ☐ **2- لا** ☐ **3- لا أتذكر**

**34) هل تم تطعيمك ضد فيروس التهاب الكبد B؟**

☐ **1- نعم** ☐ **2- لا** ☐ **3- لا أتذكر**

**35) هل تم تطعيمك كشخص بالغ؟**

☐ **1- نعم، (نوع التطعيم،________________)** ☐ **2- لا**

**36) هل تم تطعيمك ضد فيروس التهاب الكبد A كشخص بالغ؟**

☐ **1- نعم** ☐ **2- لا، ولا أخطط لذلك** ☐ **3- لا، و لكني أخطط لذلك** ☐ **4- أحتاج المزيد من المعلومات لأقرر**

37) هل لديك معلومات أو فكرة أياً من الفيروسات المرتبطة بالتهاب الكبد الوبائي تعد جزء من جدول التطعيمات في دولة الامارات العربية المتحدة ؟ ( يمكن اختيار أكثر من إجابة )

☐ 1) التهاب الكبد A ☐ 2) التهاب الكبد B ☐ 3) التهاب الكبد C
☐ 4) التهاب الكبد D ☐ 5) التهاب الكبد E ☐ 6) التهاب الكبد F
7) لا اعلم

38) ما مدى أهمية جدول التطعيمات في دولة الأمارات العربية المتحدة لك عند تطعيم أطفالك؟

| | | | | |
|---|---|---|---|---|
| 1) أبدا | 2) قليلة | 3) متوسطة | 4) كبيرة | 5) كبيرة جدا |

39) ما هي احتمالية تطعيمك لأطفالك بالمستقبل ضد فيروس التهاب الكبد A، مع العلم أن التطعيم ضده ليس إجباريا من قبل دولة الإمارات العربية المتحدة ؟

| | | | | |
|---|---|---|---|---|
| 1) أبدا | 2) قليلة | 3) متوسطة | 4) كبيرة | 5) كبيرة جدا |

40) ما هي احتمالية تطعيم طفلك إذ ما تم تصنيف فيروس التهاب الكبد A من التطعيمات الإجبارية من قبل الدولة ؟

| | | | | |
|---|---|---|---|---|
| 1) أبدا | 2) قليلة | 3) متوسطة | 4) كبيرة | 5) كبيرة جدا |

41) برأيك ، ما هي أكبر العوائق التي يمكن أن تواجه التطعيم ؟ ( يمكن أختيار أكثر من أجابه )

☐ 1) السعر ☐ 2) عدم التوفر ☐ 3) مخاوف بشأن السلامة
☐ 4) عدم معرفة الفائدة ☐ 5) أخرى، يرجى التحديد(________)

## مصادر المعرفة والمصداقية

42) إذ ما أردت التعلم أكثر عن التطعيم و الأثار الجانبية له أياً مما يلي قد يكون مرجعك الأول للحصول على المعلومات ؟ (اختار اجابه واحده )

☐ 1-طبيب عام ☐ 2- الإنترنت و مواقع التواصل الإجتماعي ☐ 3- الصيدلاني
☐ 4- الكتيبات و المنشورات ☐ 5- الأهل، الأصدقاء، و الزملاء ☐ 6- أخرى، يرجى التحديد(________)

43) إذ أردت التعلم عن مرض معين أو عن حالات طبية، أياً مما يلي قد يكون مرجعك الأول للحصول على المعلومات ؟ (اختار اجابه واحده )

☐ 1- طبيب عام ☐ 2-الإنترنت و مواقع التواصل الإجتماعي ☐ 3- الصيدلاني
☐ 4- الكتيبات و المنشورات ☐ 5-الأهل، الأصدقاء، و الزملاء ☐ 6-أخرى، يرجى التحديد(________)

44 ) هل تريد معرفة المزيد عن التهاب الكبد A ؟

☐ 1- نعم
2- لا ( انتقل لسؤال 46 )

45) إذ أردت التعلم أكثر عن مرض فيروس التهاب الكبد A، أي مما يلي قد يكون **مرجعك الأول** للحصول على المعلومات ؟ (اختار اجابه واحده )

☐ 1- طبيب عام
☐ 2-الإنترنت و مواقع التواصل الإجتماعي
☐ 3 - الصيدلاني
☐ 4- الكتيبات و المنشورات
☐ 5-الأهل، الأصدقاء، و الزملاء
☐ 6-أخرى، يرجى التحديد(________)

ما مدى مصداقية كلا من المصادر التالية ؟

| المصدر | درجة المصداقية | | | | |
|---|---|---|---|---|---|
| | 1)غير موثوق أبدا | 2) قليلا | 3) متوسط | 4) موثوق | 5) موثوق جدا |
| 46) طبيب عام | | | | | |
| 47) الإنترنت و مواقع التواصل الإجتماعي | | | | | |
| 48) الصيدلاني | | | | | |
| 49) الكتيبات و المنشورات | | | | | |
| 50) الأهل، الأصدقاء، و الزملاء | | | | | |

***شكرا للمشاركة !***
